# Influence of Electrolyte on the Electrode/Electrolyte Interface Formation on InSb Electrode in Mg-Ion Batteries

**DOI:** 10.3390/molecules26185721

**Published:** 2021-09-21

**Authors:** Irshad Mohammad, Lucie Blondeau, Jocelyne Leroy, Hicham Khodja, Magali Gauthier

**Affiliations:** 1 Université Paris-Saclay, CEA, CNRS, NIMBE, LEEL, 91191 Gif-sur-Yvette, France; lucie.patacchini@gmail.com (L.B.); hicham.khodja@cea.fr (H.K.); 2 Université Paris-Saclay, CEA, CNRS, NIMBE, LICSEN, 91191 Gif-sur-Yvette, France; jocelyne.leroy@cea.fr

**Keywords:** Magnesium-ion batteries, surface chemistry, alloys, electrode surface film, electrolytes

## Abstract

Achieving the full potential of magnesium-ion batteries (MIBs) is still a challenge due to the lack of adequate electrodes or electrolytes. Grignard-based electrolytes show excellent Mg plating/stripping, but their incompatibility with oxide cathodes restricts their use. Conventional electrolytes like bis(trifluoromethanesulfonyl)imide ((Mg(TFSI)_2_) solutions are incompatible with Mg metal, which hinders their application in high-energy Mg batteries. In this regard, alloys can be game changers. The insertion/extraction of Mg^2+^ in alloys is possible in conventional electrolytes, suggesting the absence of a passivation layer or the formation of a conductive surface layer. Yet, the role and influence of this layer on the alloys performance have been studied only scarcely. To evaluate the reactivity of alloys, we studied InSb as a model material. Ex situ X-ray photoelectron spectroscopy (XPS) and electrochemical impedance spectroscopy were used to investigate the surface behavior of InSb in both Grignard and conventional Mg(TFSI)_2_/DME electrolytes. For the Grignard electrolyte, we discovered an intrinsic instability of both solvent and salt against InSb. XPS showed the formation of a thick surface layer consisting of hydrocarbon species and degradation products from the solvent (THF) and salt (C_2_H_5_MgCl−(C_2_H_5_)_2_AlCl). On the contrary, this study highlighted the stability of InSb in Mg(TFSI)_2_ electrolyte.

## 1. Introduction

Nowadays, lithium-ion batteries (LIBs) are the main power source for portable applications due to their high energy and power density [1]. However, further development of LIBs is still restricted by limited resources, high cost, and safety issues [2]. The high demand of efficient, inexpensive, and safe electrical energy storage has accelerated the development of new battery technologies. Alternative approaches based on positive ion shuttle such as sodium-ion batteries (NIBs), magnesium-ion batteries (MIBs), and calcium-ion batteries (CIBs) have been developed. Recently, aluminum-ion batteries (AIBs) and potassium-ion batteries (KIBs) have also gained importance [3,4,5,6,7,8,9,10,11]. Among them, the electrochemical storage technology based on magnesium ion transport emerged as a promising candidate for post-lithium systems. Magnesium is an excellent alternative to lithium due to its high specific capacity, low cost, abundance on Earth, and low reactivity. In terms of volumetric capacity, Mg metal promises a relatively higher value (3833 mAh cm^−3^) than Li metal (2047 mAh cm^−3^) [12]. More importantly, the natural abundance of Mg (29 000 ppm) in the Earth’s crust is much higher compared to Li (17 ppm) [13]. Currently, the main bottleneck for the development of Mg-based technologies is the lack of a suitable electrolyte allowing both reversible Mg electrodeposition at the anode and reversible cation insertion in cathode materials at high potential. In contrast to lithium metal, the reversibility of Mg plating/stripping is limited in conventional electrolytes such as the Mg(TFSI)_2_ (magnesium bis(trifluoromethane sulfonyl)imide) salt in a diglyme solvent. A blocking passivation layer forms that prevents Mg^2+^ ion migration [14,15]. To address this issue, several strategies are being employed, most of them towards developing advanced electrolytes with wide electrochemical stability window and high ionic conductivity [16,17,18,19,20]. However, an adequate electrolyte compatible with both Mg anode and high-potential cathode is yet to be found.

Employing alternative negative electrodes based on elements forming alloys with Mg is another promising approach to enhance the viability of MIBs. The alloying/dealloying reactions of several *p*-block elements such as Sn, Sb, In, Pb, and Bi with Mg occur at a slightly higher potential (below 0.3 V vs. Mg/Mg^2+^) than the Mg plating/stripping, and with promising theoretical capacities [21,22,23,24,25,26]. Preliminary reports suggested that intermetallic anodes are compatible with a conventional electrolyte such as Mg(TFSI)_2_ in acetonitrile solution, opening the way for the use of conventional electrolytes to fabricate full MIB cells [5]. Using alloys seems thus a good strategy to avoid the surface passivation problems in standard electrolytes, without yet a clear understanding of the mechanisms at work [5,27]. Seminal questions still exist on the existence of a surface layer or on its ionically conductive or passivating nature.

Several magnesium alloys such as Mg_2_Sn and Mg_3_Bi_2_ were first investigated as negative electrodes for magnesium storage in half-cell configuration using all phenyl complex (APC) electrolytes [27,28]. Recently, Ikhe et al. demonstrated the feasibility of 3Mg/Mg_2_Sn composite as an anode for high performance MIBs in a standard electrolyte solution [28], while Blondeau et al. investigated In-based intermetallic alloys (InSb and InPb) as negative electrodes using magnesium aluminum chloride complex (MACC) electrolyte solution: EtMgCl−Et_2_AlCl in THF (Et = ethyl, THF = Tetrahydrofuran) [29,30]. In the case of InSb, the synergy created between In and Sb unlocked the electrochemical reversibility of Sb with Mg. The InSb anode, cycled in a half-cell, delivered a first magnesiation capacity of around 500 mA h g^−1^, while a capacity of 300 mAh g^−1^ was obtained in the subsequent cycles. The partial reversibility of Sb in InSb has still to be fully explained. It can be linked to the electrode microstructure or correlate with the nature of the interface formed.

The solid electrolyte interphase (SEI), which is derived from the decomposition of electrolyte at the electrode surface [31,32,33], plays a major role in cycling, power capability, and cycle life of Li-ion batteries. The SEI formation for LIBs has proven to be quite beneficial, as it allows cation diffusion while blocking electrons from the electrode, preventing further electrolyte decomposition [34]. In contrast to the large amount of investigations on the SEI formation in LIBs anodes, interfacial studies for MIB alloy electrodes are scarce [27].

The formation and evolution of the surface layer can influence strongly the batteries performance and behaviors of alloy materials. It is thus crucial to understand the mechanisms underlying its formation as well as its chemical properties to understand the performance reported. In this work, we used InSb as a model alloy and we evaluated the composition of the surface layer formed on the electrode upon reaction with Mg. The surface chemistry of InSb electrodes cycled in MACC electrolyte was examined by electrochemistry and X-ray photoelectron spectroscopy (XPS). For comparison, the chemical composition of the InSb surface was also evaluated in a Mg(TFSI)_2_-based electrolyte. 

## 2. Materials and Methods

### 2.1. Synthesis of Materials

In (99.9%), Sb (anhydrous, 99.5%), Mg (99.8%), and carbon (Csp, 99+%) were purchased from Alfa Aesar (Thermo Fisher GmbH, Kandel, Germany). Ethylmagnesium chloride [EtMgCl, 2.0 M in tetrahydrofuran (THF), diethylaluminium chloride (Et_2_AlCl, 97%), carboxymethyl cellulose (CMC), and Diethylene glycol dimethyl ether (DME) were received from Sigma-Aldrich (Saint Quentin Fallavier, France). Mg(TFSI)_2_ (Solvionic, 99.5%), salt was used after drying at 150 °C in a vacuum oven. Mg plates (99.95%) and Cu foils (12 µm) were acquired from Gallium Source and Oak Mitsui, respectively. The InSb compound was produced according to a previous report [29]. For the synthesis of InSb, a stoichiometric proportion of In and Sb was ball milled in a stainless-steel container (volume, 80 mL) containing 3 stainless steel balls (diameter, 10 mm) using a planetary-type mill (Fritsch Pulverisette, Fritsch GmbH, Idar-Oberstein, France). The ball to powder ratio was 70: 1 and milling was performed for 6 h at a speed of around 300 rpm (revolution per minute) under inert atmosphere (Ar). MgIn and Mg_3_Sb_2_ compounds were synthesized by mechanical milling in a similar fashion.

### 2.2. Electrode Preparation and Electrochemical Tests

The composite electrodes were formulated by dispersing simultaneously 80 wt% active material (i.e., InSb), 10 wt% carbon, and 10 wt% CMC binder in deionized water using a milling apparatus (MM400, Retsch, Eragny, France) for 20 min. Afterwards, the slurry was casted on Cu foils by using a spiral film coater and then dried in air for 12 h. The electrode film was punched into discs (14 mm in diameter) and then dried at 110 °C for 24 h under vacuum prior to transfer into an argon filled glove box (JACOMEX, Dagneux, France). The final mass loading of active material on the electrode was 0.65 ± 0.13 mg cm^−2^. The electrochemical tests of the as-prepared electrodes were carried out against a Mg plate as both counter and reference electrode using Swagelok-type cells (Swagelok, Villebon sur Yvette, France). The electrolyte was formulated according to previous reports and the final composition was 0.35 M EtMgCl–Et_2_AlCl in THF [29]. For comparison, an electrolyte solution consisting of 0.5 M Mg(TFSI)_2_ in DME solvent was used. Whatman glass-fibers (GF/A) (Dutscher, Bernolsheim, France) were used as separators and soaked with electrolyte. All the tests were performed at room temperature using a multichannel VMP3 potentiostat (Biologic Science Instruments, Grenoble, France) under galvanostatic mode (GCPL) between 0.005 to 0.8 V vs. Mg^2+^/Mg at a current rate of C/50. Electrochemical Impedance Spectroscopy (EIS) analyses were carried out on InSb/Mg half-cells in the frequency range of 1 MHz to 1 Hz and with a voltage amplitude of 5 mV. 

### 2.3. XPS Analysis

The composition of the surface layer was examined by ex situ XPS on InSb electrodes recovered immediately after cycling. The cells were first disassembled inside an Ar-filled glove box, the electrodes were recovered and rinsed with THF, and finally dried to guarantee that no trace of electrolyte was left. XPS measurements were carried out with a Kratos Axis Ultra DLD spectrometer (Kratos Analytical Ltd., Manchester, UK) using a monochromatic Al Kα excitation (1486.7 eV) at 150 W and a charge compensation system. The high-resolution core peaks were recorded with a constant pass energy of 40 eV. Several precautionary steps were taken to avoid the contact of samples with air and moisture. Hence, all the samples were handled under control Ar-atmosphere. A XPS transfer vessel was employed to transfer the samples from the glove box to the spectrometer. The binding energy scale was calibrated with the hydrocarbon contamination using C1s peak at 284.8 eV. A nonlinear Shirley-type background was used, while the core peaks and the corresponding areas were analyzed by a weighted least-squares fitting method using Lorentzian line shapes [35]. 

## 3. Results and Discussion

### 3.1. Electrochemical Behavior of InSb Electrode in a Grignard-Based Electrolyte

**Figure 1:**
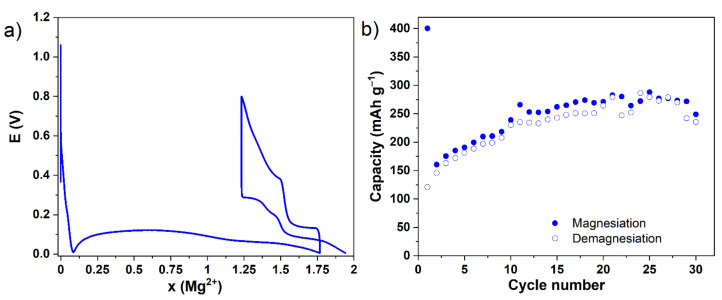
(**a**) Galvanostatic profiles and (**b**) capacities upon cycling for InSb/Mg cells cycled at C/50 rate between 0.8 and 0.005 V in 0.35 M EtMgCl–Et_2_AlCl/THF.

Figure 1a shows the galvanostatic profile for an InSb electrode cycled vs. Mg metal at a C/50 rate in a 0.35 M EtMgCl–Et_2_AlCl/THF electrolyte. A full electrochemical characterization of InSb can be found in a previous work [29]. In the first magnesiation, two regions are observed at 0.09 and 0.16 V, as expected from previous results [29]. These two regions correspond respectively to the formation of Mg_3_Sb_2_ and MgIn during the magnesiation process. In the first magnesiation, 1.8 Mg^2+^ are inserted, but only 0.5 Mg^2+^ are extracted in the first demagnesiation, indicating a poor reversibility. Figure 1b depicts the cycling performance of the InSb electrode vs. Mg at C/50. The InSb electrode exhibits a first magnesiation capacity of around 400 mAh g^−1^. This is lower than the theoretical capacity of 566 mAh g^−1^ (assuming a five electrons transfer), which denotes an incomplete reaction at the negative electrode. The first demagnesiation capacity equals to 121 mAh g ^−1^, corresponding to only 30% of the initial magnesiation capacity. In the subsequent cycles, both magnesiation and demagnesiation capacities increase slightly, while a decay occurs around 25 cycles. The earlier studies on alloy type electrode materials in MIBs [36,37,38] and LIBs [39,40] already reported a low electrochemical activity in the initial cycle. This behavior could be related to a limited use of active material in the early cycling, at the surface and near the surface region. Upon cycling, the active material reactivity seems to gradually increase from surface region to bulk through an activation process [41]. The partial reversibility of InSb could also be related to volume changes in the electrode during the first magnesiation and to the partial reversibility of the Sb/Mg_3_Sb_2_ reactions [29]. Usually, conversion type electrodes experience severe volume changes upon cycling (estimated around 100% for the InSb), leading to the disconnection of active material particles from electronic and ionic additives, resulting in capacity decay [36,37,38,39,40]. On the other hand, the irreversible capacity could be correlated to electrolyte degradation at the electrode/electrolyte interface [42]. The decomposition of electrolyte/solvent during electrochemical cycling may lead to the formation of a passivation layer containing species with poor ionic conductivity, which may impede Mg^2+^ ions migration across the layer [27].

**Figure 2:**
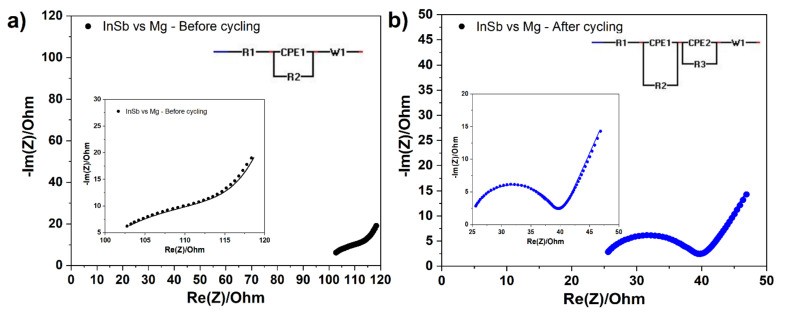
Nyquist plots obtained (**a**) before and (**b**) after cycling of InSb/Mg cells. The corresponding equivalent circuit for fitting the plots is depicted in each figure. The equivalent circuit parameters: R1, R2, R3, and W correspond to the ohmic resistance, interface resistance, charge-transfer resistance, and Warburg (cation diffusion) resistance, respectively. CPE1 and CPE2 represent constant phase elements associated with R2 and R3, respectively. The fitting factors (χ^2^/|Z|) obtained from the EIS before and after cycling are 0.012 and 0.013, respectively.

Generally, electronic and ionic segregation of active material at the electrode surface could lead to an increase of the cell resistance, and be responsible for the capacity fading [43]. To get insights into the irreversibility mechanism of the InSb electrode, EIS measurements were performed on InSb/Mg half-cells. Nyquist plots obtained before and after cycling along with corresponding equivalent circuits used for fitting the data are given in Figure 2a and 2b. The fitted values are given in Table 1. Before cycling, the cell spectrum consists of a highly depressed semicircle at high frequency and a straight line at low frequency, corresponding respectively to interface resistance and Warburg impedance [44]. After 30 cycles, the spectrum consists of two overlapped semicircles and a tilted straight line. The additional semicircle is attributed to charge-transfer resistance. The total resistance of the cell (R = R1 + R2 + R3) decreases after cycling (Table 1), suggesting that the internal resistance of the cell is not responsible for the capacity fading. Before cycling, the ohmic resistance (R1) is 98 Ω, and decreases to 24 Ω after cycling, due to the process of adjusting the internal components: the infiltration of electrolyte inside the electrode, the distribution of electrode materials, and the compact relation of transport processes to electrode structures. However, the interfacial resistance (R2) seems to increase slightly after the charge-discharge cycles, denoting the formation of a surface film by electrolyte decomposition that gradually thickens by the accumulation of decomposition products during cycling. After cycling, appearance of charge transfer resistance (R3) indicates that electrochemical activity occurred at the electrode surface. Charge transfer resistance (R3) is linked to the process of electron transfer from one phase to another. Based on the overall cell resistance, it becomes evident that the global cell resistance is not responsible for the low reversible capacity. Some other factors such as the passivation layer formed by decomposition of electrolyte and solvent and electronic and ionic segregation of active material within the electrode could be the reason for poor reversible capacity, which possibly came from volume changes of the electrode [45,46]. To understand better the effect of the surface layer on the electrochemical behavior of InSb electrodes, XPS analyses were performed to investigate the evolution of the surface layer composition in two different electrolyte solutions.

### 3.2. Chemical Composition and Evolution of the InSb Electrode Surface

#### 3.2.1. Surface Layer Composition in Grignard-Based Electrolyte

**Figure 3:**
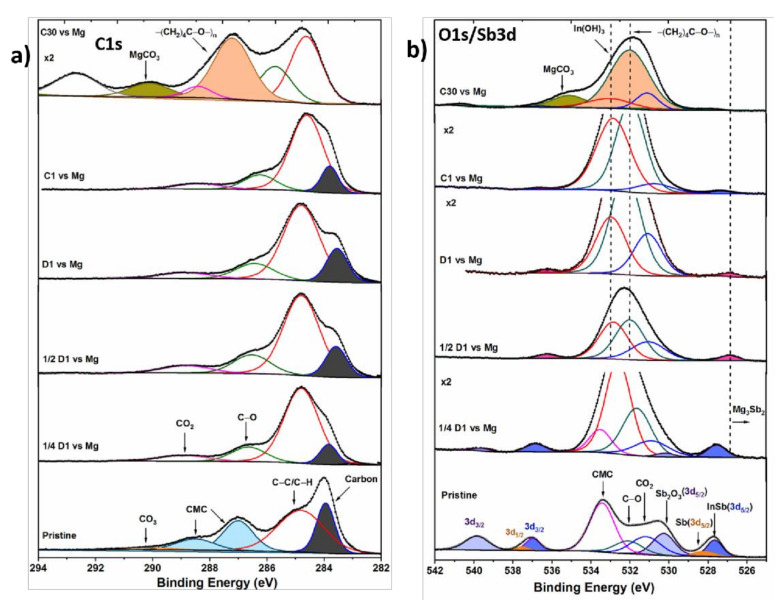
(**a**) C1s and (**b**) O1s/Sb3d core peaks spectra of the InSb electrode cycled in EtMgCl–Et2AlCl/THF electrolyte vs. Mg. ¼D1, ½D1, D1, C1, and C30 denote the first one-quarter discharge, first half discharge, first discharge, first charge, and 30th charge, respectively. All spectra were calibrated by considering the adventitious carbon peak (C-C/C-H) at 284.8 eV.

**Figure 4:**
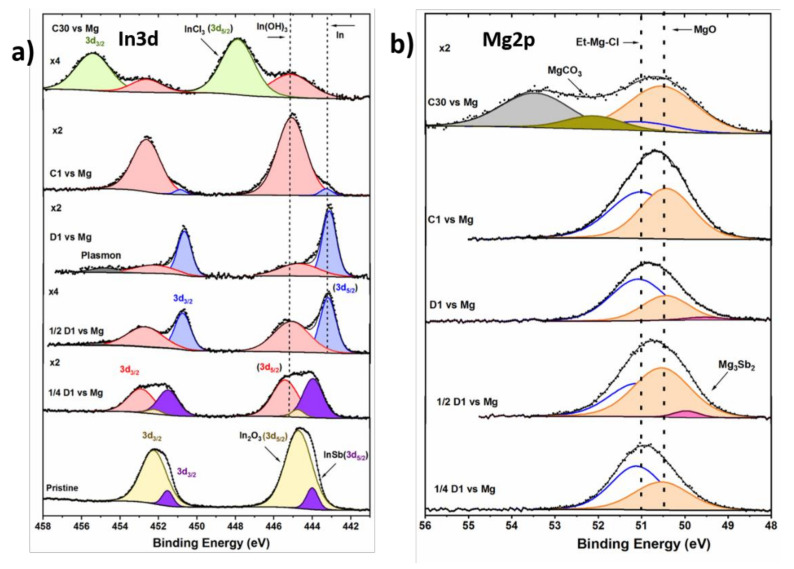
(**a**) In3d and (**b**) Mg 2p core peaks spectra of the InSb electrode cycled in EtMgCl–Et2AlCl/THF electrolyte vs. Mg. All spectra were calibrated by considering the adventitious carbon peak (C-C/C-H) at 284.8 eV.

**Figure 5:**
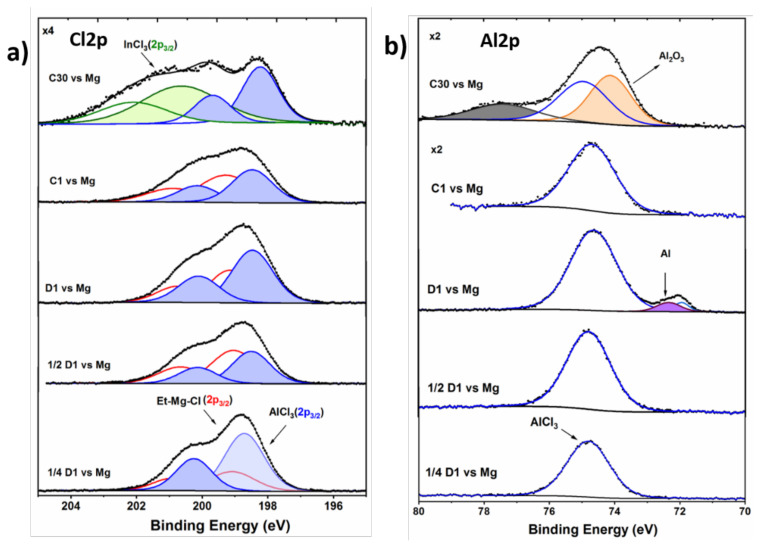
(**a**) Cl2p and (**b**) Al2p core peaks spectra of the InSb electrodes cycled in EtMgCl–Et_2_AlCl/THF electrolyte vs. Mg.

The composition of the InSb electrode surface in a Grignard-based electrolyte was investigated by ex situ XPS measurements. The C1s, O1s/Sb3d, In3d, Mg2p, Al2p, and Cl2p spectra of pristine, discharged, and charged InSb electrodes are shown in Figure 3, Figure 4 and Figure 5. They correspond to electrodes cycled at different states of the first discharge and charge and after a prolonged cycling of 30 cycles.

- C1s region

Figure 3a displays the XPS C1s core spectra obtained for pristine and cycled InSb electrodes in the EtMgCl–Et_2_AlCl/THF electrolyte. For the pristine InSb composite electrode, the C1s core spectrum shows three major components related to the carbon additive, aliphatic chains (C-C/C-H), and the CMC binder [47,48]. A weak peak corresponding to CO_3_ is observed at a high binding energy (290.0 eV) [49]. In the beginning of discharge (one-quarter of discharge ¼D1), the peaks linked to the CMC binder are no longer detected, suggesting the formation of a layer on the electrode surface whose thickness is exceeding the penetration limit of XPS measurement (5–10 nm). Yet, the peak related to carbon additives is still observed, implying that the surface layer thickness is smaller than 5–10 nm, allowing the detection of a part of the carbon additive. It can also be noticed that the carbon peak is slightly shifted towards lower binding energy for the discharged samples, as previously reported [50]. In addition, new carbonaceous species corresponding to C-O and CO_2_ environments appear at the surface of cycled electrodes. After completing half of the first discharge and the full first discharge (½D1 and D1), a similar pattern with some relative intensity fluctuations is observed, denoting a continuous thickening of the surface film. For the first charge (C1), no changes are observed in the electrode surface composition, but that trend changes after prolonged cycling. In the C30 spectrum, corresponding to an electrode discharged and charged for 30 cycles, the carbon additive signal is no longer detected, revealing the formation of a much thicker surface film. Two new important features appear at the surface: MgCO_3_ (290.8 eV) and -(CH_2_)_4_C-O-)_n_ (287.8 eV) species. The existence of carbonate species in the XPS spectrum is obvious for the electrode recovered after a long cycling, in agreement with earlier reports [41,51]. The signal of polyether -(CH_2_)_4_C-O-)_n_ is highly intense and corresponds to degradation products from the THF solvent. The relative concentrations of all the components of the C1s spectra are gathered in Table S1.

- Sb3d/O1s region

The Sb3d/O1s core spectrum of the pristine InSb electrode (Figure 3b) presents three sets of doublets corresponding to the active material InSb, Sb oxide, and unreacted metallic Sb [52,53,54,55]. The minor Sb component is related to traces of unreacted Sb during the InSb synthesis, likely to remain at the topmost surface. The prominent peak of the spectrum located at 533.4 eV is assigned to the O1s contribution of the CMC binder. At the ¼D1 stage, the initial components observed on the pristine electrode slightly decrease in intensity, suggesting the formation of a thin surface layer. In contrast to the pristine sample, here the intensity of Sb_2_O_3_ is relatively lower than InSb, implying that some portion of oxidized Sb participates in the magnesiation reaction, as already observed for surface tin oxides [37,41], or reacts with the electrolyte. Surprisingly, a broad peak corresponding to In(OH)_3_ is observed around 533.0 eV), which might arise from a parasitic and irreversible chemical reaction between discharged InSb and the THF solvent [56,57]. On further discharge (½D1 and D1), all the peaks related to pristine components vanish, while a new doublet corresponding to the Mg_3_Sb_2_ (526.8, 536.2 eV) compound appears (reference spectra of Mg_3_Sb_2_ are given in Figure S1). The presence of Mg_3_Sb_2_ illustrates the alloying reaction of Mg with Sb, in agreement with previous electrochemical and XRD data [29]. Interestingly, the Sb3d peaks corresponding to the InSb active material reappear in the charge spectrum (C1), demonstrating the reversibility of the magnesiation reactions. The electrode surface after one charge (C1) possesses the same composition as the discharged sample surface layer in the O1s region, denoting the growth of the surface layer. After prolonged cycling (C30), the O1s spectrum clearly evidences changes in the surface composition, where two additional species are detected: MgCO_3_ and -(CH_2_)_4_C-O-)_n_, verifying the findings of the C1s spectrum for the C30 sample. In this case, the surface species observed on the first cycle are hardly detected due to the formation of a relatively much thicker surface film. A continuous increase in the content of oxide species is observed upon cycling (Table S1), especially the C-O associated component, which increased to 12% (from 4.7%) after the first discharge (D1), evidencing the steady degradation of the solvent and electrolyte molecules.

- In3d region

Figure 4a illustrates the XPS In3d core peak spectra for the InSb electrode at the different stages of (de)magnesiation. Two sets of doublets corresponding to InSb and In_2_O_3_ components are observed for the pristine electrode [54,58,59]. At the beginning of discharge (¼D1), a new feature like In(OH)_3_ is observed (along with In_2_O_3_ and InSb), revealing again the chemical reactivity between the electrode and the electrolyte/solvent. After the first discharge, the decrease of the In_2_O_3_ peak intensity suggests its involvement in the magnesiation process or its reaction with the electrolyte. In the ½D1 spectrum, a new set of peaks fitting with In metal is detected (reference spectra of In in Figure S1), which arises from the extrusion of In from the InSb alloy, as already seen in the magnesiation and lithiation of InSb [29,60]. Unlike the electrochemical profile, MgIn is not identified in the In3d spectrum, as confirmed by the Mg2p spectrum (Figure 4b). This could be related to the overlapping of MgIn and In peaks as both show similar binding energies (Figure S1). On further magnesiation (D1), the intensity of the metallic In peaks increases, while the peaks vanish upon charging (C1). This may underline the reversibility of the demagnesiation process to form back the InSb compound as seen in the Sb3d spectra, or a reaction of metallic In with the electrolyte solvent to form In(OH)_3_. In any case, the large amount of In(OH)_3_ detected suggests that this species might be a byproduct of a chemical reaction between the active material and the solvent, revealing the instability of InSb in Grignard type electrolytes. To support this hypothesis, deeper study about interfacial chemistry of the InSb is required. After prolonged cycling (C30 sample), InCl_3_ is observed as a new specie in the surface layer [61,62]. Its formation may be the consequence of EtMgCl decomposition, that may further react with InSb or In metal via Cl^−^ transfer, as suggested in earlier reports on alloys [63,64].

- Mg2p region

The XPS Mg2p core spectra (Figure 4b) for all discharged (¼D1, ½D1, and D1) and charged samples (C1) are characterized mainly by two components: EtMgCl and MgO [37,64]. The EtMgCl corresponds to the electrolyte salt traces remaining at the surface despite the rinsing of the electrode with THF solvent. MgO appears as the main component of the Mg-based species. Its presence might originate from the electrolyte decomposition. For ½D1 and D1 samples, a minor peak at 49.8 eV is assigned to Mg_2_Sb_3_ (reference spectra of Mg_3_Sb_2_ in Figure S1), as already featured in the discharge O1s/Sb3d spectrum (Figure 3b). In the C30 spectrum, after prolonged cycling, magnesium carbonate MgCO_3_ is detected at 52.1 eV, in agreement with the O1s and C1s spectra (Figure 3). The presence of MgCO_3_ is mainly due to electrolyte decomposition. Finally, another peak appears at a high binding energy of 53.5 eV, but was not identified. It might be related to other Mg-based degradation products.

- Cl2p and Al2p regions

Finally, Cl and Al-based species were detected on the electrode surface as shown in the Cl2p and Al2p XPS spectra in Figure 5a and b. All the samples (except C30) show similar species in various concentrations (Figure 5a). Two sets of doublets corresponding to EtMgCl and AlCl_3_ (as confirmed by Al2p spectrum) are observed on the Cl2p spectra. Both species can be attributed to residual Cl-based species from the as-synthesized electrolyte. The concentration of AlCl_3_ seems to be relatively higher in comparison to EtMgCl in all the cases, indicating the constant decomposition of the electrolyte. In the deconvoluted spectrum of the C30 sample, a new component attributed to InCl_3_ is observed, as already verified from the In3d spectrum (Figure 4a). In the D1 sample, traces of metallic Al are detected, which arise most likely from the reduction of Al anions to Al^0^ during discharge [65]. During the first charge, the intensity of the AlCl_3_ compound decreases significantly, highlighting a partial dissolution of the species. For the C30 sample, an Al signal is detected at 74.1 eV and is probably assigned to Al_2_O_3_, which can result from the oxidation of some Al species during prolonged cycling [65].

#### 3.2.2. Surface Layer Composition in a Mg(TFSI)_2_-Based Electrolyte

**Figure 6:**
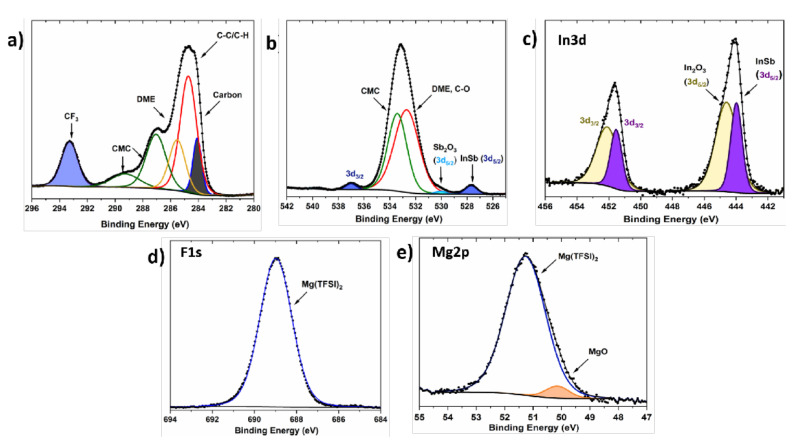
(**a**) C1s, (**b**) O1s/Sb3d, (**c**) In3d, (**d**) F1s, and (**e**) Mg2p core peaks spectra of slightly demagnesiated InSb in 0.5 M Mg(TFSI)_2_/DME electrolyte.

To compare the reactivity of the alloy compound in the Grignard electrolyte with a conventional electrolyte, we investigated the surface layer formed on a InSb electrode cycled in a Mg(TFSI)_2_-based electrolyte. Figure 6a–d shows the C1s, O1s/Sb3d, In3d, F1s, and Mg2p spectra for a InSb electrode cycled in 0.5 M Mg(TFSI)_2_/DME electrolyte and slightly discharged (Figure S2). The Mg metal counter electrode in this system is quickly passivated in the Mg(TFSI)_2_/DME electrolyte, leading to a limited stripping of Mg metal, and thus explaining the limited number of Mg^2+^ that reacts with the InSb material. Therefore, the cycling mainly denotes the reactivity of InSb with the electrolyte and the formation of the surface layer.

The C1s spectrum of the discharged electrode in Figure 6a consists of five carbonaceous species corresponding to carbon additive, adventitious hydrocarbon, CMC binder, DME, and CF_3_ (electrolyte) [66]. The presence of CF_3_ is related to the Mg(TFSI)_2_ salt residue remaining at the surface, as confirmed by the Mg 2p and F1s spectra (Figure 6d–e) [27]. The appearance of CMC and carbon additive signal with decreased intensity suggests that a thin surface layer is formed on the discharged electrode. The O1s spectrum verifies the existence of CMC and C-O containing species (DME and adventitious carbon) at the electrode surface. The deconvoluted Sb3d and In3d spectra (Figure 6b–c) show the presence of the active material InSb and respective metal oxides (Sb and In). A significant decrease in the amount of surface oxidized metal species compared to the pristine electrode (Figure 3b, bottom layer) reveals their involvement in the magnesiation process, in consistence with what has been observed for the electrode discharged (¼ D1) in the Grignard-based electrolyte. Unlike the EtMgCl–Et_2_AlCl case, magnesiated products like Mg_3_Sb_2_ and metallic In are not observed here, probably due to the limited magnesiation of the electrode.

Interestingly, the nature of the chemical species formed on the electrode surface in Mg(TFSI)_2_ electrolyte is completely different as compared to the EtMgCl–Et_2_AlCl case. No compounds such as In(OH)_3_ are observed on the surface, in contrast to what was revealed on the surface formed in the Grignard electrolyte at the early stage of discharge. This suggests a better stability of the InSb material in the conventional electrolyte. Overall, the spectra evidenced the limited degradation of the DME (C_6_H_14_O_3_) solvent as well as the TFSI^−^ (C_2_FNO_4_S_2_) anion during the cycling of the InSb, which contrasts with the observation for the corrosive electrolyte, where decomposition of both salt and solvent was observed. This suggests that the electrolyte and solvent degradation is minimal in this case and the surface layer is very thin, revealing a significant stability of the InSb electrode in the conventional electrolyte. This study seems to confirm the better stability of InSb in the conventional electrolyte, predicting its applicability as an anode material in full high-voltage MIB cells.

#### 3.2.3. Discussion

The XPS results presented herein denote the evolution of the chemical compositions formed at the InSb surface cycled in two different solutions: the corrosive EtMgCl-Et_2_AlCl/THF electrolyte and the conventional Mg(TFSI)_2_/DME electrolyte. After (de)magnesiation, a typical composition consisting of hydrocarbon species, carbonates, and degradation products of solvents and salts is detected on the electrode surface in the Grignard electrolyte. Several carbonaceous species such as aliphatic chains, adventitious carbon oxides (C-O and CO_2_), and carbonate are observed. During initial cycling, a thin surface layer is formed on the electrode, which eventually allows the detection of some part of the active material electrode underneath the surface layer. The electrochemical mechanism of InSb (de)alloying is also evidenced by the XPS spectra. The magnesiation of InSb forms Mg_2_Sb_3_ and In, and demagnesiation of Mg_2_Sb_3_ and In transfers back to InSb, indicating a reversible electrochemical process. Unfortunately, the magnesiation product MgIn was not clearly evidenced as its characteristic binding energies are very close to metallic In. A prolonged cycling in the Grignard electrolyte leads to the formation of a thicker surface layer (≥5 nm), revealing a continuous growth of the surface layer and an extended electrolyte degradation upon cycling. This extended decomposition might arise from the repeated volume changes upon (de)magnesiation that create fresh surfaces for electrolyte interfacial reactions. The presence of species such as In(OH)_3_, MgO, EtMgCl, and AlCl_3_ at the electrode/electrolyte interface indicates that the EtMgCl-Et_2_AlCl electrolyte decomposes through chemical reactions. After long cycling, more decomposition products like carbonates, polyether, and In chloride are detected, representing a steady decomposition of the electrolyte upon cycling. As a comparison, the interfacial behavior of InSb in the early stage of cycling was revealed in a conventional electrolyte. The nature of the surface layer on InSb in a Mg(TFSI)_2_-based electrolyte is completely changed and the reactivity of the DME solvent seems marginal with a very thin surface layer deposited on the electrode surface. In contrast to Grignard electrolyte, the XPS analysis suggests fortunately a limited reactivity in this electrolyte concerning the InSb material, predicting its applicability as anode in high-voltage Mg batteries. This stresses out the importance of the electrolyte’s nature in the interfacial reactivity of alloys materials. 

## 4. Conclusions

In the present study, we investigated with ex situ XPS the surface reactivity of the InSb material at different stages of cycling in two different electrolytes for Mg batteries. The surface chemistry of the InSb alloy in a Grignard-type electrolyte evidenced the continuous growth of a surface layer upon cycling. The surface layer is constituted mostly of organic and inorganic compounds from both the electrolyte salt and solvent degradation such as carbon-oxygen species, MgO, AlCl_3_, and indium hydroxides. In contrast, after 30 cycles the surface composition changes drastically with the building of a thicker layer mainly composed of MgCO_3_, polyether chains derived from the solvent, and In chloride, highlighting the high reactivity of the InSb alloy compound in the Grignard electrolyte. 

While the cycling of the InSb intermetallic compound shows the formation of a thick decomposition layer in Grignard electrolyte, the reactivity seems limited in a conventional electrolyte based on the Mg(TFSI)_2_ salt. This discrepancy in the reactivity with electrolytes may affect the global performance of the alloy. The high reactivity of the alloys in the Grignard-based electrolyte may consume electrons and ions and cause irreversibility. For sake of simplicity, most studies on alloys for Mg batteries evaluate the performance of alloys in half-cell using a Grignard electrolyte to allow the use of metallic Mg as counter and reference electrodes. However, we showed herein that this can be misleading. This may lead scientists to conclude wrongly that the alloy performance is low, while increased performance can be obtained in a more suitable electrolyte. This study highlights how crucial the choice of electrolyte is in the performance’s evaluation of alloy compounds for Mg batteries and calls for caution in the anode/electrolyte selection to obtain the more accurate view of the electrochemical and chemical processes in these promising materials.

## Figures and Tables

**Table 1:** Fitting parameters of the Nyquist plots for InSb/Mg cells.

InSb/Mg Cell	Before Cycling	After Cycling
R1 (Ω)	98	24
R2 (Ω)	6	12
R3 (Ω)	−	3
W (Ω s^−1/2^)	3300	100
CPE1 (F s^a−1^)	0.56 × 10^−6^	3.6 × 10^−6^
CPE2 (F s^a−1^)	−	2 × 10^−4^

## Data Availability

Data are contained within the article.

## Supplementary Materials

The following are available online. Table S1: XPS atomic percentages of the chemical species formed on the InSb electrode in the Grignard electrolyte. Figure S1: (a) The C1s, (b) O1s/Sb3d, (c) In, and (d) Mg 2p core peaks spectra of pure In, MgIn, and Mg_3_Sb_2_ powders. Figure S2: Galvanostatic profile for a InSb/Mg cell cycled at C/100 rate in 0.5 M Mg(TFSI)_2_/DME.

## Sample Availability

Samples of the compounds are not available from the authors.

## References

[1] Choi J.W., Aurbach D. (2016). Promise and Reality of Post-Lithium-Ion Batteries with High Energy Densities. Nat. Rev. Mater..

[2] Yaksic A., Tilton J.E. (2009). Using the Cumulative Availability Curve to Assess the Threat of Mineral Depletion: The Case of Lithium. Resour. Policy.

[3] Hasa I., Mariyappan S., Saurel D., Adelhelm P., Koposov A.Y., Masquelier C., Croguennec L., Casas-Cabanas M. (2021). Challenges of Today for Na-Based Batteries of the Future: From Materials to Cell Metrics. J. Power Sources.

[4] Ellis B.L., Nazar L.F. (2012). Sodium and Sodium-Ion Energy Storage Batteries. Curr. Opin. Solid State Mater. Sci..

[5] Dominko R., Bitenc J., Berthelot R., Gauthier M., Pagot G., Di Noto V. (2020). Magnesium Batteries: Current Picture and Missing Pieces of the Puzzle. J. Power Sources.

[6] Aurbach D., Lu Z., Schechter A., Gofer Y., Gizbar H., Turgeman R., Cohen Y., Moshkovich M., Levi E. (2000). Prototype Systems for Rechargeable Magnesium Batteries. Nature.

[7] Gummow R.J., Vamvounis G., Kannan M.B., He Y. (2018). Calcium-Ion Batteries: Current State-of-the-Art and Future Perspectives. Adv. Mater..

[8] Ponrouch A., Frontera C., Bardé F., Palacín M.R. (2016). Towards a Calcium-Based Rechargeable Battery. Nat. Mater..

[9] Wu X., Leonard D.P., Ji X. (2017). Emerging Non-Aqueous Potassium-Ion Batteries: Challenges and Opportunities. Chem. Mater..

[10] Eftekhari A. (2004). Potassium Secondary Cell Based on Prussian Blue Cathode. J. Power Sources.

[11] Lei X., Zheng Y., Zhang F., Wang Y., Tang Y. (2020). Highly stable magnesium-ion-based dual-ion batteries based on insoluble small-molecule organic anode material. Energy Storage Mater..

[12] Yoo H.D., Shterenberg I., Gofer Y., Gershinsky G., Pour N., Aurbach D. (2013). Mg Rechargeable Batteries: An On-Going Challenge. Energy Environ. Sci..

[13] Orikasa Y., Masese T., Koyama Y., Mori T., Hattori M., Yamamoto K., Okado T., Huang Z.-D., Minato T., Tassel C. (2014). High Energy Density Rechargeable Magnesium Battery Using Earth-Abundant and Non-Toxic Elements. Sci. Rep..

[14] Lossius L.P., Emmenegger F. (1996). Plating of Magnesium from Organic Solvents. Electrochim. Acta.

[15] Lu Z., Schechter A., Moshkovich M., Aurbach D. (1999). On the Electrochemical Behavior of Magnesium Electrodes in Polar Aprotic Electrolyte Solutions. J. Electroanal. Chem..

[16] Zhao-Karger Z., Bardaji M.E.G., Fuhr O., Fichtner M. (2017). A New Class of Non-Corrosive, Highly Efficient Electrolytes for Rechargeable Magnesium Batteries. J. Mater. Chem. A.

[17] Mizrahi O., Amir N., Pollak E., Chusid O., Marks V., Gottlieb H., Larush L., Zinigrad E., Aurbach D. (2007). Electrolyte Solutions with a Wide Electrochemical Window for Rechargeable Magnesium Batteries. J. Electrochem. Soc..

[18] Kim H.S., Arthur T.S., Allred G.D., Zajicek J., Newman J.G., Rodnyansky A.E., Oliver A.G., Boggess W.C., Muldoon J. (2011). Structure and Compatibility of a Magnesium Electrolyte with a Sulphur Cathode. Nat. Commun..

[19] Barile C.J., Spatney R., Zavadil K.R., Gewirth A.A. (2014). Investigating the Reversibility of in Situ Generated Magnesium Organohaloaluminates for Magnesium Deposition and Dissolution. J. Phys. Chem. C.

[20] Son S.-B., Gao T., Harvey S.P., Steirer K.X., Stokes A., Norman A., Wang C., Cresce A., Xu K., Ban C. (2018). An Artificial Interphase Enables Reversible Magnesium Chemistry in Carbonate Electrolytes. Nat. Chem..

[21] Singh N., Arthur T.S., Ling C., Matsui M., Mizuno F. (2012). A High Energy-Density Tin Anode for Rechargeable Magnesium-Ion Batteries. Chem. Commun..

[22] Arthur T.S., Singh N., Matsui M. (2012). Electrodeposited Bi, Sb and Bi_1-x_Sb_x_ Alloys as Anodes for Mg-Ion Batteries. Electrochem. Commun..

[23] Murgia F., Weldekidan E.T., Stievano L., Monconduit L., Berthelot R. (2015). First Investigation of Indium-Based Electrode in Mg Battery. Electrochem. Commun..

[24] Periyapperuma K., Tran T.T., Purcell M.I., Obrovac M.N. (2015). The Reversible Magnesiation of Pb. Electrochim. Acta.

[25] Shao Y., Gu M., Li X., Nie Z., Zuo P., Li G., Liu T., Xiao J., Cheng Y., Wang C. (2014). Highly Reversible Mg Insertion in Nanostructured Bi for Mg Ion Batteries. Nano Lett..

[26] Niu J., Zhang Z., Aurbach D. (2020). Alloy Anode Materials for Rechargeable Mg Ion Batteries. Adv. Energy Mater..

[27] Matsui M., Kuwata H., Mori D., Imanishi N., Mizuhata M. (2019). Destabilized Passivation Layer on Magnesium-Based Intermetallics as Potential Anode Active Materials for Magnesium Ion Batteries. Front. Chem..

[28] Ikhe A.B., Han S.C., Prabakar S.J.R., Park W.B., Sohn K.-S., Pyo M. (2020). 3Mg/Mg_2_Sn Anodes with Unprecedented Electrochemical Performance towards Viable Magnesium-Ion Batteries. J. Mater. Chem. A.

[29] Blondeau L., Foy E., Khodja H., Gauthier M. (2019). Unexpected Behavior of the InSb Alloy in Mg-Ion Batteries: Unlocking the Reversibility of Sb. J. Phys. Chem. C.

[30] Blondeau L., Surblé S., Foy E., Khodja H., Gauthier M. (2021). Electrochemical Reactivity of In-Pb Solid Solution as a Negative Electrode for Rechargeable Mg-Ion Batteries. J. Energy Chem..

[31] Heiskanen S.K., Kim J., Lucht B.L. (2019). Generation and Evolution of the Solid Electrolyte Interphase of Lithium-Ion Batteries. Joule.

[32] Fong R., von Sacken U., Dahn J.R. (1990). Studies of Lithium Intercalation into Carbons Using Nonaqueous Electrochemical Cells. J. Electrochem. Soc..

[33] Peled E., Menkin S. (2017). Review—SEI: Past, Present and Future. J. Electrochem. Soc..

[34] An S.J., Li J., Daniel C., Mohanty D., Nagpure S., Wood D.L. (2016). The State of Understanding of the Lithium-Ion-Battery Graphite Solid Electrolyte Interphase (SEI) and Its Relationship to Formation Cycling. Carbon.

[35] Shirley D.A. (1972). High-Resolution X-Ray Photoemission Spectrum of the Valence Bands of Gold. Phys. Rev. B.

[36] Wang L., Welborn S.S., Kumar H., Li M., Wang Z., Shenoy V.B., Detsi E. (2019). High-Rate and Long Cycle-Life Alloy-Type Magnesium-Ion Battery Anode Enabled Through (De)Magnesiation-Induced Near-Room-Temperature Solid–Liquid Phase Transformation. Adv. Energy Mater..

[37] Nguyen D.-T., Song S.-W. (2017). Magnesium Stannide as a High-Capacity Anode for Magnesium-Ion Batteries. J. Power Sources.

[38] Xu X., Chao D., Chen B., Liang P., Li H., Xie F., Davey K., Qiao S.-Z. (2020). Revealing the Magnesium-Storage Mechanism in Mesoporous Bismuth via Spectroscopy and Ab-Initio Simulations. Angew. Chem. Int. Ed..

[39] Hong S., Jo H., Song S.-W. (2015). Lithium Diffusivity of Tin-Based Film Model Electrodes for Lithium-Ion Batteries. J. Electrochem. Sci. Technol..

[40] Liu Y., Wang L., Jiang K., Yang S. (2019). Electro-Deposition Preparation of Self-Standing Cu-Sn Alloy Anode Electrode for Lithium Ion Battery. J. Alloys Compd..

[41] Nguyen G.T.H., Nguyen D.-T., Song S.-W. (2018). Unveiling the Roles of Formation Process in Improving Cycling Performance of Magnesium Stannide Composite Anode for Magnesium-Ion Batteries. Adv. Mater. Interfaces.

[42] Delpuech N., Dupré N., Mazouzi D., Gaubicher J., Moreau P., Bridel J.-S., Guyomard D. (2013). Lestriez, B. Correlation between irreversible capacity and electrolyte solvents degradation probed by NMR in Si-based negative electrode of Li-ion cell. Electrochem. Commun..

[43] Mandli A.R., Kaushik A., Patil R.S., Naha A., Hariharan K.S., Kolake S.M., Han S., Choi W. (2019). Analysis of the effect of resistance increase on the capacity fade of lithium ion batteries. Energy Res..

[44] Talaie E., Bonnick P., Sun X., Pang Q., Liang X., Nazar L.F. (2017). Methods and Protocols for Electrochemical Energy Storage Materials Research. Chem. Mater..

[45] Kumar R., Tokranov A., Sheldon B.W., Xiao X., Huang Z., Li C., Mueller T. (2016). In Situ and Operando Investigations of Failure Mechanisms of the Solid Electrolyte Interphase on Silicon Electrodes. ACS Energy Lett..

[46] Attias R., Salama M., Hirsch B., Goffer Y., Aurbach D. (2019). Anode-Electrolyte Interfaces in Secondary Magnesium Batteries. Joule.

[47] Pantea D., Darmstadt H., Kaliaguine S., Roy C. (2003). Electrical Conductivity of Conductive Carbon Blacks: Influence of Surface Chemistry and Topology. Appl. Surf. Sci..

[48] Pantea D., Darmstadt H., Kaliaguine S., Sümmchen L., Roy C. (2001). Electrical Conductivity of Thermal Carbon Blacks: Influence of Surface Chemistry. Carbon.

[49] El Ouatani L., Dedryvère R., Ledeuil J.-B., Siret C., Biensan P., Desbrières J., Gonbeau D. (2009). Surface Film Formation on a Carbonaceous Electrode: Influence of the Binder Chemistry. J. Power Sources.

[50] Philippe B., Dedryvère R., Allouche J., Lindgren F., Gorgoi M., Rensmo H., Gonbeau D., Edström K. (2012). Nanosilicon Electrodes for Lithium-Ion Batteries: Interfacial Mechanisms Studied by Hard and Soft X-Ray Photoelectron Spectroscopy. Chem. Mater..

[51] Aswal D.K., Muthe K.P., Tawde S., Chodhury S., Bagkar N., Singh A., Gupta S.K., Yakhmi J.V. (2002). XPS and AFM Investigations of Annealing Induced Surface Modifications of MgO Single Crystals. J. Cryst. Growth.

[52] Madec L., Gachot G., Coquil G., Martinez H., Monconduit L. (2018). Toward Efficient Li-Ion Cells at High Temperatures: Example of TiSnSb Material. J. Power Sources.

[53] Bodenes L., Darwiche A., Monconduit L., Martinez H. (2015). The Solid Electrolyte Interphase a Key Parameter of the High Performance of Sb in Sodium-Ion Batteries: Comparative X-Ray Photoelectron Spectroscopy Study of Sb/Na-Ion and Sb/Li-Ion Batteries. J. Power Sources.

[54] Li W., Liu T., Zhang J., Peng N., Zheng R., Yu H., Bai Y., Cui Y., Shu J. (2019). Commercially Available InSb as a High-Performance Anode for Secondary Batteries towards Superior Lithium Storage. Sustain. Energy Fuels.

[55] Mohammad I., Blondeau L., Foy E., Leroy J., Leroy E., Khodja H., Gauthier M. (2021). Nanostructured Intermetallic InSb as a High-Capacity and High-Performance Negative Electrode for Sodium-Ion Batteries. Sustain. Energy Fuels.

[56] Teterin Y.A., Maslakov K.I., Murav’ev E.N., Teterin A.Y., Bulychev N.A., Meshkov B.B., Stepnov D.S. (2020). X-Ray Photoelectron Spectroscopy Study of Indium Tin Mixed Oxides on the Surface of Silicate Glass. Inorg. Mater..

[57] Loh J.Y.Y., Kherani N.P. (2019). X-Ray Photospectroscopy and Electronic Studies of Reactor Parameters on Photocatalytic Hydrogenation of Carbon Dioxide by Defect-Laden Indium Oxide Hydroxide Nanorods. Molecules.

[58] Copperthwaite R.G., Kunze O.A., Lloyd J., Neely J.A., Tuma W. (1978). Surface Analysis of InSb by X-Ray Photoelectron Spectroscopy (XPS). Z. Nat. A.

[59] Sahu S., Manivannan A., Shaik H., Mohan Rao G. (2017). Local Structure of Amorphous Ag_5_In_5_Sb_60_Te_30_ and In_3_SbTe_2_ Phase Change Materials Revealed by X-Ray Photoelectron and Raman Spectroscopic Studies. J. Appl. Phys..

[60] Hieu L.T., So S., Kim I.T., Hur J. (2021). Highly Reversible Lithiation/Delithiation in Indium Antimonide with Hybrid Buffering Matrix. Int. J. Energy Res..

[61] Hu Y., Zhou D.-Y., Wang B., Wang Z.-K., Liao L.-S. (2016). Chlorinated Indium Tin Oxide Electrode by InCl_3_ Aqueous Solution for High-Performance Organic Light-Emitting Diodes. Appl. Phys. Lett..

[62] Berestok T., Guardia P., Portals J.B., Estrade S., Llorca J., Peiro J., Cabot A., Brock S.L. (2018). Surface Chemistry and Nano-/Microstructure Engineering on Photocatalytic In_2_S_3_ Nanocrystals. Langmuir.

[63] Aurbach D., Weissman I., Gofer Y., Levi E. (2003). Nonaqueous Magnesium Electrochemistry and Its Application in Secondary Batteries. Chem. Rec..

[64] Nguyen D.-T., Tran X.M., Kang J., Song S.-W. (2016). Magnesium Storage Performance and Surface Film Formation Behavior of Tin Anode Material. ChemElectroChem.

[65] Wang Z., Bandyopadhyay A., Kumar H., Li M., Venkatakrishnan A., Shenoy V.B., Detsi E. (2019). Degradation of Magnesium-Ion Battery Anodes by Galvanic Replacement Reaction in All-Phenyl Complex Electrolyte. J. Energy Storage.

[66] Jay R., Tomich A.W., Zhang J., Zhao Y., De Gorostiza A., Lavallo V., Guo J. (2019). Comparative Study of Mg(CB_11_H_12_)_2_ and Mg(TFSI)_2_ at the Magnesium/Electrolyte Interface. ACS Appl. Mater. Interfaces.

