# Supplementary material for: Influence of Electrolyte on the Electrode/Electrolyte Interface Formation on InSb Electrode in Mg-Ion Batteries
Source: Molecules. 2021 Sep 21;26(18):5721. doi: 10.3390/molecules26185721 (PMC8472600; doi:10.3390/molecules26185721)
Supplement: Supplementary file 1 [file molecules-26-05721-s001.zip › molecules-1365072-supplementary.pdf]

Influence of Electrolyte on the Electrode/Electrolyte Interface Formation on InSb Electrode in Mg-Ion Batteries

Irshad Mohammad 1,, Lucie Blondeau 1, Jocelyne Leroy 2, Hicham Khodja 1 and Magali Gauthier 1,**

1 Université Paris-Saclay, CEA, CNRS, NIMBE, LEEL, 91191 Gif-sur-Yvette, France;

lucie.patacchini@gmail.com (L.B.); hicham.khodja@cea.fr (H.K.)

2 Université Paris-Saclay, CEA, CNRS, NIMBE, LICSEN, 91191 Gif-sur-Yvette, France; joce-

lyne.leroy@cea.fr

* Correspondence: irshad.mohammad636@gmail.com (I.M.); magali.gauthier@cea.fr

(M.G.); Tel.: +33-169-0845-30 (M.G.)

Table S1. XPS atomic percentages of the chemical species formed on the InSb electrode in the Grignard electrolyte.

Component		Binding Energy (eV)	Atomic percentage of the component (%)					
			Pristine	¼ D1	½ D1	D1	C1	C30
Sb3d	InSb	527.7-537.0	0.5	0.2	-	-	< 0.1	-
	Sb	528.2-537.5	< 0.1	-	-	-	-	-
	Sb_2O_3	530.3-539.8	0.8	< 0.1	-	-		-
	Mg_3Sb_2	526.9-536.2	-	-	0.13	0.05		-
O1s	CO_2/MO	531.0	3.6	2.5	5.0	4.5	1.6	3.0
	C-O	532.0	4.7	6.0	9.0	11.9	11.0	-
	CMC	533.4	11.3	2.3	-	-	-	-
	$In(OH)_3$	533.0	-	10.8	8.4	6.7	10.7	3.8
	$-(CH_2)_4C-O-)_n$		-	-	-	-	-	18.7
	$MgCO_3$		-	-	-	-	-	3.5
C1s	Carbon additive	284.0	19.0	5.5	7.8	8.1	5.7	-
	C-C/C-H	284.8	30.8	40.0	37.3	35.1	31.0	13.6
	CO	287.0	17.4	7.8	8.7	7.0	11.0	10.7
	CO_2	288.5	6.4	4.5	4.5	3.8	5.9	4.0
	CO_3	290.0	2.4	-	-	-	-	-
	$-(CH_2)_4C-O-)_n$		-	-	-	-	-	12.5
	$MgCO_3$		-	-	-	-	-	4.0
In3d	InSb	444.0-451.5	0.2	0.3	-	-	-	-
	In_2O_3	444.7-452.2	1.5	< 0.1	-	-		-
	$In(OH)_3$	445.1-452.6	-	0.3	0.3	0.1	0.8	0.1
	In	443.1-450.6	-	-	0.3	0.1	< 0.1	-
	$InCl_3$	447.9	-	-	-	-		0.3
Mg2p	EtMgCl	51.1	-	3.6	2.3	3.6	5.2	0.5
	MgO	50.5	-	2.7	4.5	1.9	4.8	2.8
	Mg_3Sb_2	49.9	-	-	0.4	0.2	-	-
	$MgCO_3$		-	-	-	-	-	0.7
Cl2p	EtMgCl	199.0-200.7	-	1.4	2.5	2.2	2.5	-
	$AlCl_3$	198.7-200.0	-	5.1	2.0	3.6	2.8	1.0
	$InCl_3$		-	-	-	-	-	1.3
Al2p	$AlCl_3$	74.8	-	6.3	5.1	8.4	3.0	2.1
	Al	72.1	-	-	-	0.8	-	
	Al_2O_3	74.1	-	-	-	-	-	2.2

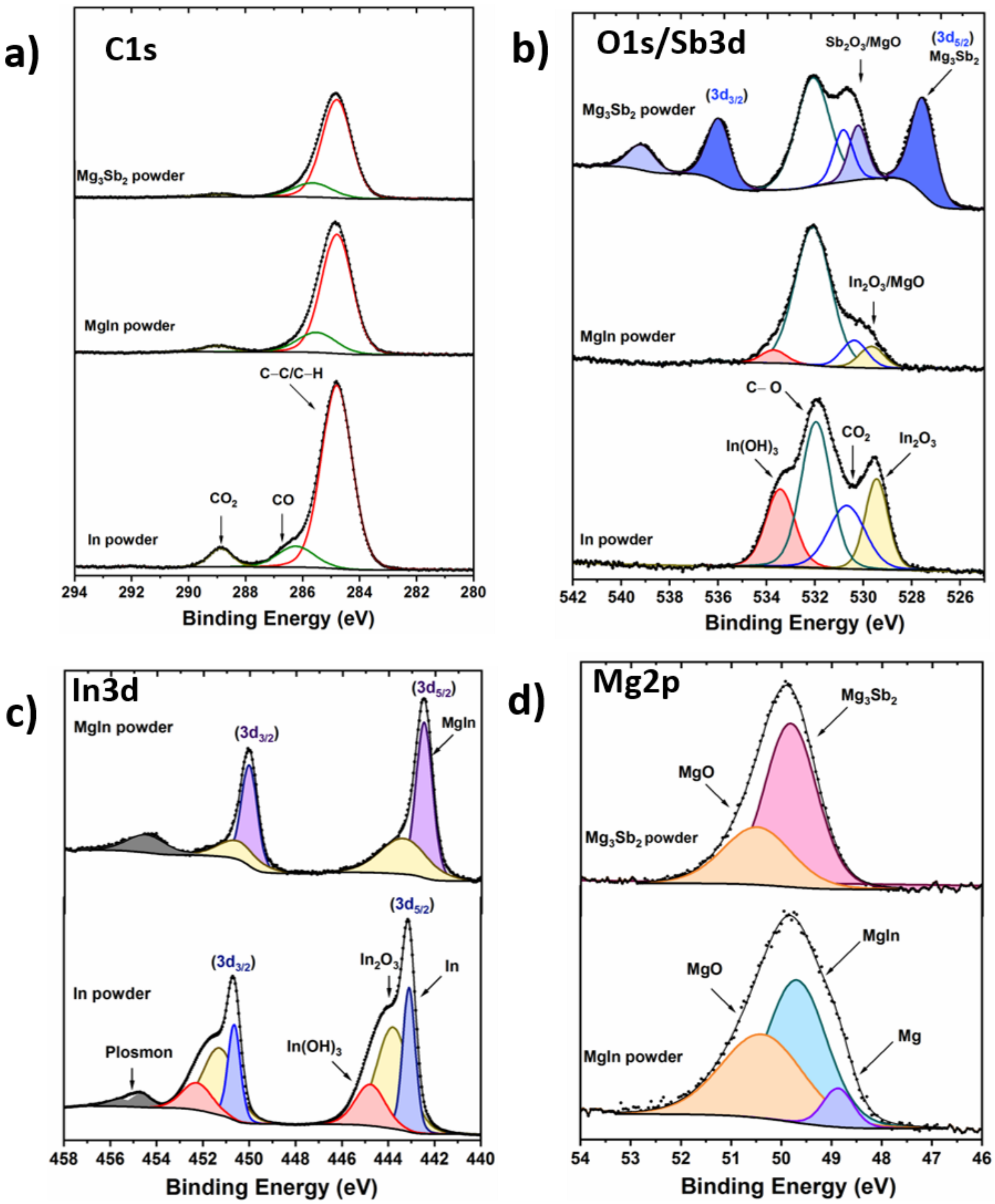

Figure S1. (a) The C1s, (b) O1s/Sb3d, (c) In, and (d) Mg 2p core peaks spectra of pure In, MgIn, and Mg_3Sb_2 powders.

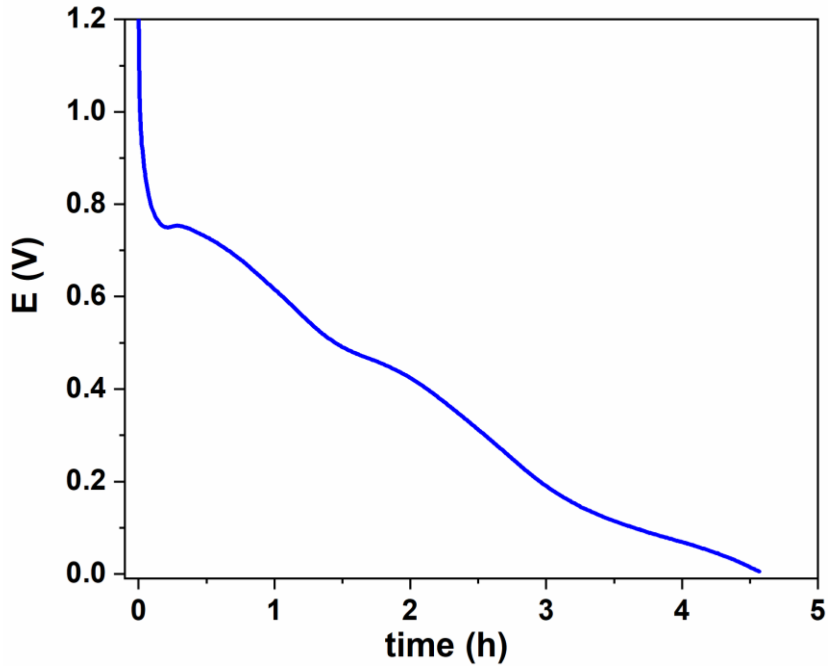

Figure S2. Galvanostatic profile for a InSb/Mg cell cycled at C/100 rate in 0.5 M $Mg(TFSI)_2$/DME.
